# Web-Based Search Volume for HIV Tests and HIV-Testing Preferences During the COVID-19 Pandemic in Japan: Infodemiology Study

**DOI:** 10.2196/52306

**Published:** 2024-01-18

**Authors:** Rie Kanamori, Futaba Umemura, Kosuke Uemura, Taiju Miyagami, Simon Valenti, Nobuyuki Fukui, Mayumi Yuda, Mizue Saita, Hirotake Mori, Toshio Naito

**Affiliations:** 1 Department of General Medicine Faculty of Medicine Juntendo University Tokyo Japan; 2 Department of Sports Medicine and Sportology Graduate School of Medicine Juntendo University Tokyo Japan; 3 Center for Promotion of Data Science Graduate School of Medicine Juntendo University Tokyo Japan

**Keywords:** HIV test, infodemiology, self-test, COVID-19, search engine, Japan

## Abstract

**Background:**

Research has found a COVID-19 pandemic–related impact on HIV medical services, including clinic visits, testing, and antiviral therapy initiation in countries including Japan. However, the change in trend for HIV/AIDS testing during the COVID-19 pandemic has not been explored extensively in the Japanese population.

**Objective:**

This infodemiology study examines the web-based search interest for two types of HIV tests, self-test kits and facility-based tests, before and during the COVID-19 pandemic in Japan.

**Methods:**

The monthly search volume of queried search terms was obtained from Yahoo! JAPAN. Search volumes for the following terms were collected from November 2017 to October 2018: “HIV test,” “HIV test kit,” and “HIV test health center.” The search term “Corona PCR” and the number of new COVID-19 cases by month were used as a control for the search trends. The number of new HIV cases in the corresponding study period was obtained from the AIDS Trend Committee Quarterly Report from the AIDS Prevention Foundation.

**Results:**

Compared to the search volume of “corona-PCR,” which roughly fluctuated corresponding to the number of new COVID-19 cases in Japan, the search volume of “HIV test” was relatively stable from 2019 to 2022. When we further stratified by the type of HIV test, the respective web-based search interest in HIV self-testing and facility-based testing showed distinct patterns from 2018 to 2022. While the search volume of “HIV test kit” remained stable, that of “HIV test health center” displayed a decreasing trend starting in 2018 and has remained low since the beginning of the COVID-19 pandemic. Around 66%-71% of the search volume of “HIV test kits” was attributable to searches made by male internet users from 2018 to 2022, and the top three contributing age groups were those aged 30-39 (27%-32%), 20-29 (19%-32%), and 40-49 (19%-25%) years. On the other hand, the search volume of “HIV test health centers” by male users decreased from more than 500 from 2018 to 2019 to fewer than 300 from 2020 to 2022.

**Conclusions:**

Our study found a notable decrease in the search volume of “HIV test health center” during the pandemic, while the search volume for HIV self-testing kits remained stable before and during the COVID-19 crisis in Japan. This suggests that the previously reported COVID-19–related decrease in the number of HIV tests mostly likely referred to facility-based testing. This sheds light on the change in HIV-testing preferences in Japan, calling for a more comprehensive application and regulatory acceptance of HIV self-instructed tests.

## Introduction

HIV prevalence in Japan is <0.1% among adults aged 15-49 years, and the cumulative total of people diagnosed and living with HIV at the end of 2018 was approximately 30,000 [1]. A recent study using a national database found that 81.5% of people living with HIV who were on antiretroviral therapy (ART) had at least one chronic comorbidity [2]. According to recent national statistics, the proportion of people diagnosed and living with HIV on ART was 86% and 80% as of 2018 [3], lower than the first two 90-90-90 Joint United Nations Programme on HIV and AIDS (UNAIDS) goals, while the proportion of people living with HIV on ART who are virally suppressed was as high as 99% as of 2020 [4], reaching the UNAIDS goal. Evidence has suggested that earlier HIV diagnosis and linkage to treatment should be the core strategy in controlling the HIV epidemic [5]; however, the sudden and ongoing COVID-19 pandemic has overwhelmed the health care system globally and caused severe disruptions in medical service provision, ranging from elective procedures to routine surveillance for manageable diseases such as HIV infections [6-11]. Additionally, social distancing measures implemented early in the pandemic affected people’s access or willingness to visit health care facilities.

There are concerns that disruptions caused by the COVID-19 pandemic may lead to increased HIV incidence and mortality and pose challenges to the international community’s goal to eliminate the HIV or AIDS epidemic by 2030 [1]. Reports from Africa, Asia, Europe, Japan, and the United States have found a pandemic-related impact on HIV medical services, including clinic visits, testing, and antiviral therapy initiation [1-6]. In particular, the number of HIV tests conducted in health centers in Japan reduced from 142,000 in 2019 to 69,000 in 2020, with the difference amounting to a 50% decline in the first year of the COVID-19 pandemic [3]. The number of HIV consultations performed in public health centers also significantly declined in the second quarter of 2020 (32,565 tests) compared to the same period the year before (11,689 tests) [2].

In Japan, voluntary HIV testing is free of charge and anonymous, and offered as a package service bundled with pre- and posttest counseling at appointed public health facilities by law. Although the number of health center–conducted HIV tests increased steadily and peaked in 2008, a 30% decline was observed before the COVID-19 pandemic [8,12]. This is possibly due to the commercial availability of HIV self-tests in Japan, with the number of dry blood spot HIV self-tests increasing from 26,000 tests in 2005 to 91,000 tests in 2016 [7,12]. However, the HIV self-testing technique is not yet approved by the health authorities in Japan.

The internet is a common source of disease- and health-related information, and internet use influences care initiation and the treatment decisions of people living with HIV [13]. During the COVID-19 pandemic, infodemiology studies found that web-based search trends for symptoms associated with COVID-19 coincided with the disease outbreak [14]. Conversly, the global web-based interest in information on HIV/AIDS care services decreased [15]. However, the search engine trend for HIV/AIDS testing during the COVID-19 pandemic has not been explored extensively in the Japanese population. This study aims to characterize and compare the trend for web-based search interest in HIV testing before and during the pandemic to investigate whether there is a change in HIV/AIDS health-seeking behavior in Japan.

Data from the search engine provided by the Yahoo Japan Corporation will be used for this study because Yahoo! JAPAN is the most visited website in the country, and compared to most other countries, Japan uses Google less frequently.

## Methods

### Data Source

To examine the pattern of web-based search interests in HIV testing before and during the COVID-19 pandemic, the monthly search volume of selected search terms was determined based on the number of searches over a specified period extracted from Yahoo! JAPAN, which is one of the most used digital services in Japan, and compared to the rest of the world, Google is used less. According to the data, 68.9% of the population (aged >2 years) used Yahoo! JAPAN at least once a month between January and November 2021, while 65.1% used Google in the same period [16]. Search volume data were retrieved with authorized access from the Yahoo Japan Corporation server via the DS.INSIGHT People portal. It has been used in studies focusing on transition or trends in search behavior over time among different demographics (eg, gender, age, or prefecture) [17]. The user manual is available on the web [18].

The internet user population is the number of internet users throughout Japan, calculated based on the “Telecommunications Usage Trends Survey” published by the Ministry of Internal Affairs and Communications.

The quarterly number of new COVID-19 cases between January 2019 and December 2022 was obtained from an excerpt from the Japan Ministry of Health, Labour and Welfare [19].

The number of newly infected people with HIV between December 31, 2018, and June 26, 2022, was obtained from the AIDS Trend Committee Quarterly Report from the AIDS Prevention Foundation, API-Net AIDS Prevention Information Net [20].

### Search Queries Used in the Analysis

The search term “HIV検査” (ie, “HIV test” in Japanese) was used to assess the web-based interest in general HIV testing from January 2020 to October 2021. The result is presented together with the search volume of “コロナPCR” (ie, “corona-PCR” in Japanese) and the number of new COVID-19 cases by month. The search volumes of “HIV検査キット” (ie, “HIV test kit” in Japanese) and “HIV検査 保健所” (ie, “HIV test health center” in Japanese) were compared to analyze the difference in web-based interest in self-instructed/postal and facility-based HIV testing from November 2018 to October 2022.

### Data Standardization

The monthly number of searches per prefecture for each search query was obtained, adjusted by sex, and converted to standardized *z* scores according to the following formula:




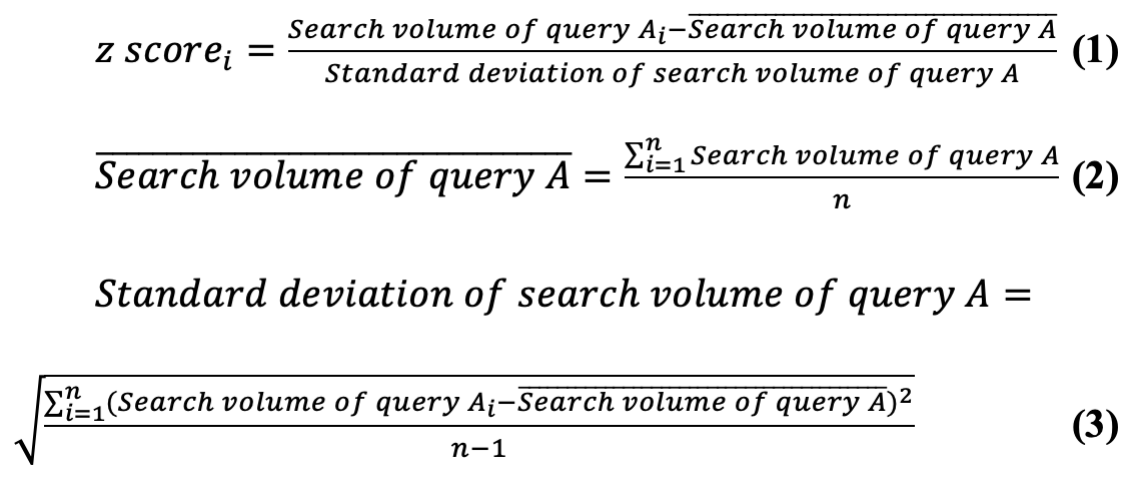




“Query A” refers to the queried search term.

### Statistical Analysis

Internet users were stratified according to age on the day of the search, sex, and year. Demographics were summarized as the number and percentage of patients for categorical variables. Descriptive statistics were summarized using Excel (Microsoft Corporation).

### Ethical Considerations

This infodemiology study adhered to chapter 1, section 3, part 1, subsection (C), item 3 of the ethical guidelines for Medical and Health Research Involving Human Subjects of the Ministry of Health, Labour and Welfare of Japan [21]. In accordance with this guideline, since this study used previously anonymized and deidentified data, an ethical review was waived, and patient informed consent was not required.

## Results

**Figure 1:**
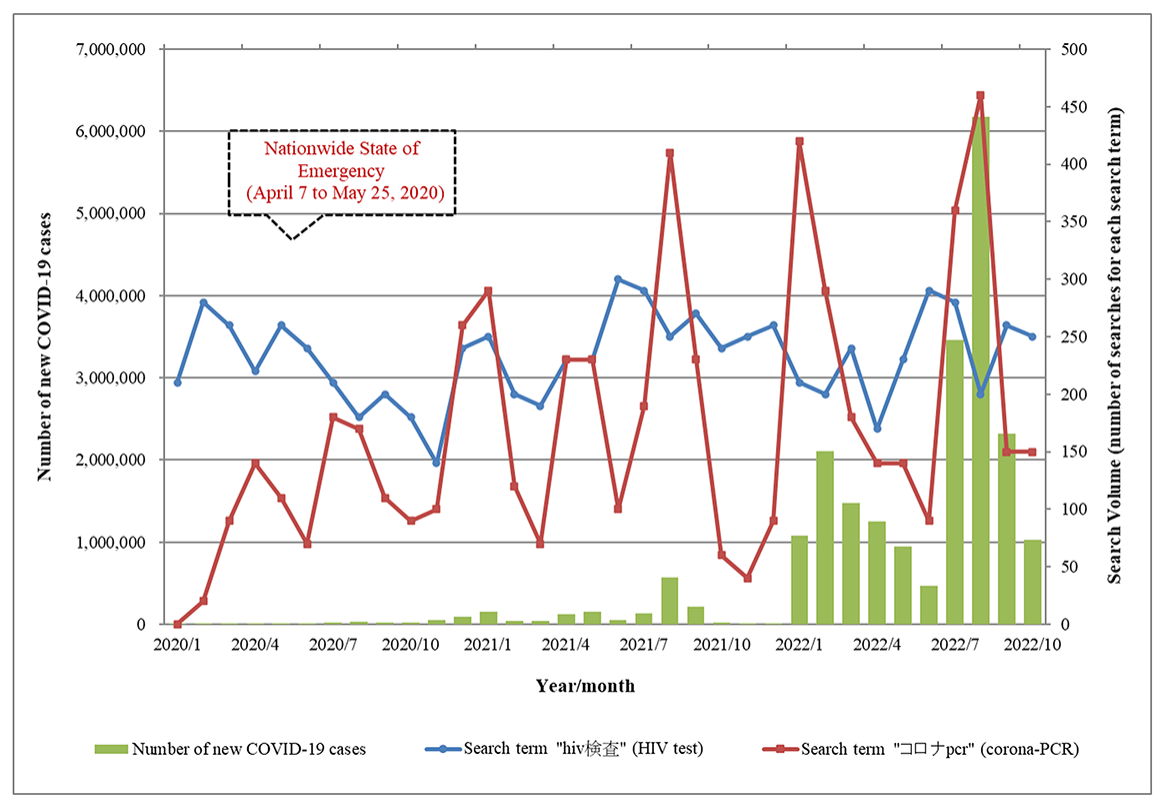
Monthly web-based search interest in HIV testing and COVID-19 genetic testing and the number of new COVID-19 cases from 2020 to 2022. The monthly search volumes of search terms “HIV検査” (HIV test in Japanese, blue line) and “コロナPCR” (corona-PCR in Japanese, red line) from January 2020 to October 2022 according to the search engine Yahoo! JAPAN are shown. The number of new COVID-19 cases in the corresponding months, according to the Ministry of Health, Labour and Welfare in Japan, is also shown in green bars in this figure.

Between 2018 and 2022, 50% to 51% of overall adult internet users were male, and the user population was evenly distributed across different age groups by decade (11%-18% across different age groups in 2018, 12%-17% in 2019, 12%-18% in 2020, 13%-17% in 2021, and 13%-17% in 2022; Tables 1-3). The first nationwide state of emergency concerning COVID-19 was from April 7 to May 25, 2020, in Japan; since then, the increase in web-based interest in COVID-19 polymerase chain reaction testing (represented by the search volume of “corona-PCR”) roughly corresponded to the peaks and troughs of new COVID-19 infection cases in recent waves of the COVID-19 pandemic in Japan during mid 2021, early 2022, and late 2022. In contrast, the web-based interest in general HIV testing represented by the search term “HIV test” appeared to be relatively stable (Figure 1). Although the sex distribution of the “corona-PCR” search volume varied between 2020 and 2022, people aged 40-49 years consistently showed the highest web-based interest (26%-27%) for COVID-19 polymerase chain reaction testing, followed by those aged 50-59 years (21%-23%) and 30-39 years (15%-18%). In line with the characteristics of people with an increased risk of HIV infection, 57%-60% of the “HIV test” search volume between 2020 and 2022 were from male internet users, and people aged between 20 and 29 years (30%-31%) showed the most interest in general HIV testing within this period, followed by those aged between 30 and 39 years (24%-27%) and 40 and 49 years (17%-20%; Table 1).

**Table 1:** Search volumes of “HIV test” and “corona-PCR” by the sex and age of internet users in the years 2020, 2021, and 2022.

			Year 2020, n (%)							Year 2021, n (%)							Year 2022 (January to October), n (%)				
			Internet user^a^		Search term “HIV検査”^b^		Search term “コロナpcr”^c^		Internet user^a^			Search term “HIV検査”		Search term “コロナpcr”		Internet user^d^			Search term “HIV検査”		Search term “コロナpcr”
Overall			96,688,079 (100)		2500 (100)		1300 (100)		99,499,669 (100)			2700 (100)		2000 (100)		99,499,669 (100)			2330 (100)		2380 (100)
**Sex**																					
	Male	49,119,275 (51)		1500 (60)		640 (50)		50,286,908 (51)			1600 (59)		820 (41)		50,286,908 (51)			1320 (57)		910 (38)	
	Female	47,568,804 (49)		980 (40)		640 (50)		49,212,761 (49)			1100 (41)		1200 (59)		49,212,761 (49)			1010 (43)		1470 (62)	
**Age group (year)**																					
	<20	13,386,518 (14)		170 (7)		20 (2)		13,787,372 (14)			280 (10)		100 (5)		13,787,372 (14)			150 (6)		110 (5)	
	20-29	11,777,729 (12)		770 (30)		130 (10)		12,432,946 (13)			840 (31)		280 (14)		12,432,946 (13)			700 (30)		350 (15)	
	30-39	13,547,255 (14)		670 (27)		190 (15)		13,615,333 (14)			680 (25)		310 (16)		13,615,333 (14)			570 (24)		430 (18)	
	40-49	17,429,796 (18)		470 (19)		340 (26)		17,491,018 (17)			460 (17)		540 (27)		17,491,018 (17)			470 (20)		610 (26)	
	50-59	15,451,255 (16)		230 (9)		300 (23)		16,258,923 (16)			250 (9)		440 (22)		16,258,923 (16)			240 (10)		510 (21)	
	60-69	12,716,666 (13)		130 (5)		190 (15)		12,887,755 (13)			130 (5)		230 (12)		12,887,755 (13)			130 (6)		200 (8)	
	≥70	12,378,860 (13)		70 (3)		110 (9)		13,026,322 (13)			80 (3)		80 (4)		13,026,322 (13)			70 (3)		170 (7)	

^a^The internet user population is the number of internet users throughout Japan, calculated based on the “Telecommunications Usage Trends Survey” published by the Ministry of Internal Affairs and Communications [21].

^b^HIV検査: HIV test (in Japanese).

^c^コロナpcr: corona-PCR (in Japanese).

^d^The internet user population in 2022 uses the figures for 2021.

**Table 2:** Search volumes for “HIV test kit” and “HIV test health center” by the sex and age of the internet users by year (2018-2020).

			Year 2018, n (%)							Year 2019, n (%)							Year 2020, n (%)				
			Internet user population^a^		Search term “hiv検査キット”^b^		Search term “hiv検査 保健所”^c^		Internet user population^a^			Search term “hiv検査キット”		Search term “hiv検査 保健所”		Internet user population^a^			Search term “hiv検査キット”		Search term “hiv検査 保健所”
Overall			95,590,219 (100)		780 (100)		940 (100)		107,480,629 (100)			850 (100)		960 (100)		96,688,079 (100)			790 (100)		580 (100)
**Sex**																					
	Male	48,932,966 (51)		540 (69)		530 (56)		53,862,486 (50)			560 (66)		540 (56)		49,119,275 (51)			560 (71)		280 (48)	
	Female	46,657,253 (49)		240 (31)		410 (44)		53,618,143 (50)			290 (34)		420 (44)		47,568,804 (49)			230 (29)		300 (52)	
**Age group (years)**																					
	<20	12,760,379 (13)		20 (3)		30 (3)		13,710,441 (13)			30 (4)		30 (3)		13,386,518 (14)			30 (4)		50 (9)	
	20-29	12,381,162 (13)		200 (26)		280 (30)		12,514,936 (12)			220 (26)		310 (33)		11,777,729 (12)			210 (26)		160 (27)	
	30-39	14,321,086 (15)		250 (31)		300 (32)		14,167,245 (13)			250 (28)		270 (28)		13,547,255 (14)			210 (27)		140 (24)	
	40-49	18,127,686 (18)		190 (24)		180 (19)		18,214,081 (16)			160 (19)		190 (20)		17,429,796 (18)			180 (23)		90 (16)	
	50-59	14,891,256 (16)		90 (12)		90 (10)		15,903,690 (15)			100 (12)		80 (8)		15,451,255 (16)			90 (11)		60 (10)	
	60-69	12,991,502 (14)		30 (4)		40 (4)		14,688,962 (14)			60 (7)		60 (6)		12,716,666 (13)			40 (5)		50 (9)	
	≥70	10,117,148 (11)		0 (0)		20 (2)		18,281,274 (17)			30 (4)		20 (2)		12,378,860 (13)			30 (4)		30 (5)	

^a^The internet user population is the number of internet users throughout Japan, calculated based on the “Telecommunications Usage Trends Survey” published by the Ministry of Internal Affairs and Communications [22].

^b^hiv検査キット: HIV test kit (in Japanese).

^c^hiv検査 保健所: HIV test health center (in Japanese).

**Table 3:** Search volumes for “HIV test kit” and “HIV test health center” by the sex and age of the internet users by year (2021 and 2022).

		Year 2021, n (%)				Year 2022 (January to October), n (%)		
		Internet user population^a^	Search term “hiv検査キット”^b^	Search term “hiv検査 保健所”^c^	Internet user population (2021)^d^		Search term “hiv検査キット”	Search term “hiv検査 保健所”
Overall		99,499,669 (100)	900 (100)	640 (100)	99,499,669 (100)		570 (100)	280 (100)
**Sex**								
	Male	50,286,908 (51)	590 (66)	290 (45)	50,286,908 (51)		390 (68)	100 (36)
	Female	49,212,761 (49)	310 (34)	350 (55)	49,212,761 (49)		180 (32)	180 (64)
**Age group (years)**								
	<20	13,787,372 (14)	30 (3)	50 (8)	13,787,372 (14)		40 (7)	30 (11)
	20-29	12,432,946 (13)	280 (32)	230 (35)	12,432,946 (13)		110 (19)	90 (32)
	30-39	13,615,333 (14)	270 (30)	170 (27)	13,615,333 (14)		180 (32)	90 (32)
	40-49	17,491,018 (17)	190 (21)	110 (17)	17,491,018 (17)		140 (25)	40 (14)
	50-59	16,258,923 (16)	90 (10)	60 (9)	16,258,923 (16)		80 (14)	20 (7)
	60-69	12,887,755 (13)	30 (3)	10 (2)	12,887,755 (13)		0 (0)	0 (0)
	≥70	13,026,322 (13)	10 (1)	10 (2)	13,026,322 (13)		20 (4)	10 (4)

^a^The internet user population is the number of internet users throughout Japan, calculated based on the “Telecommunications Usage Trends Survey” published by the Ministry of Internal Affairs and Communications [22].

^b^hiv検査キット: HIV test kit (in Japanese).

^c^hiv検査 保健所: HIV test health center (in Japanese).

^d^The internet user population in 2022 uses the figures for 2021.

**Figure 2:**
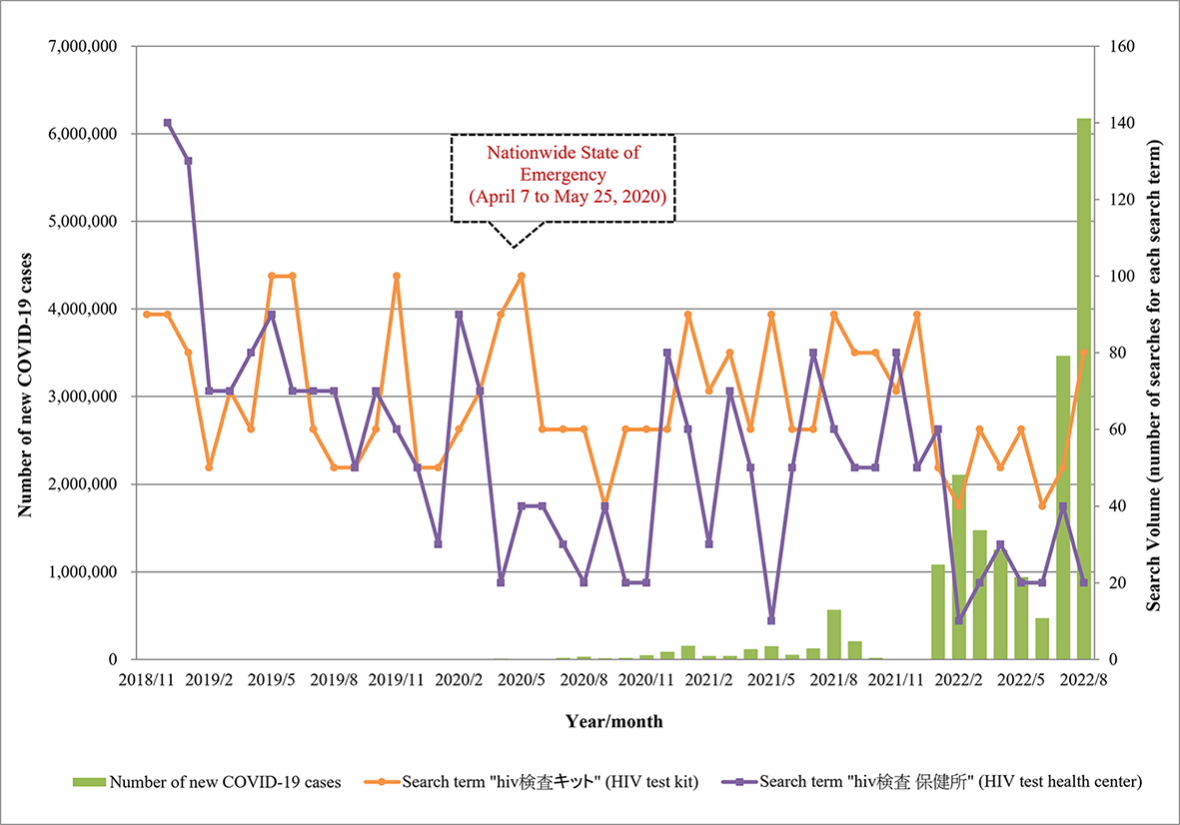
Monthly web-based search interest in HIV self-instructed and facility-based tests and the number of new COVID-19 cases from 2018 to 2022. The monthly search volumes of search terms “HIV検査キット” (HIV test kit in Japanese, orange line) and “HIV検査 保健所” (HIV test health center in Japanese, purple line) from November 2018 to October 2022 according to the search engine Yahoo! JAPAN are shown. The number of new COVID-19 cases in the corresponding months, according to the Ministry of Health, Labour and Welfare in Japan, is shown with green bars.

Web-based interest in HIV testing was further refined as either facility-based HIV tests or self-instructed HIV-testing kits. The search volume for HIV facility-based testing was high in late 2018 and decreased in several instances over the next 3 years, especially in 2020 and 2022 (Figure 2). The search volume of “HIV test kit” (used as a representative search term for HIV self-testing) was lower than that for HIV facility-based testing at the end of 2018. However, the interest remained high over the next 4 years relative to facility-based testing, particularly during the COVID-19 pandemic (Figure 2). Around 66%-71% of the search volume of “HIV test kit” was attributable to searches made by male internet users between 2018 and 2022, and the top three contributing age groups were those 30-39 years (27%-32%), 20-29 years (19%-32%), and 40-49 years (19%-25%). The overall search volume of HIV test health centers decreased from 950 in 2018/2019 to 580 in 2020. The actual search volume for “HIV test kit” versus “HIV test health center” was 560 versus 280 among male internet users in 2020 and 590 versus 290 in 2021; while among female internet users the actual search volume for “HIV test kit” versus “HIV test health center” were similar: 230 versus 300 in 2020 and 310 versus 350 in 2021. Hence, the main population searching for “HIV test health center” shifted from male to female users (n=300, 52%) in 2020 and the following years (n=350, 55% in 2021 and n=180, 64% in 2022). Internet users aged 20-29 years (27%-35%), 30-39 years (24%-32%), and 40-49 years (14%-20%) were the top three representative age groups for the search volume of “HIV test health center” (Tables 2 and 3).

**Figure 3:**
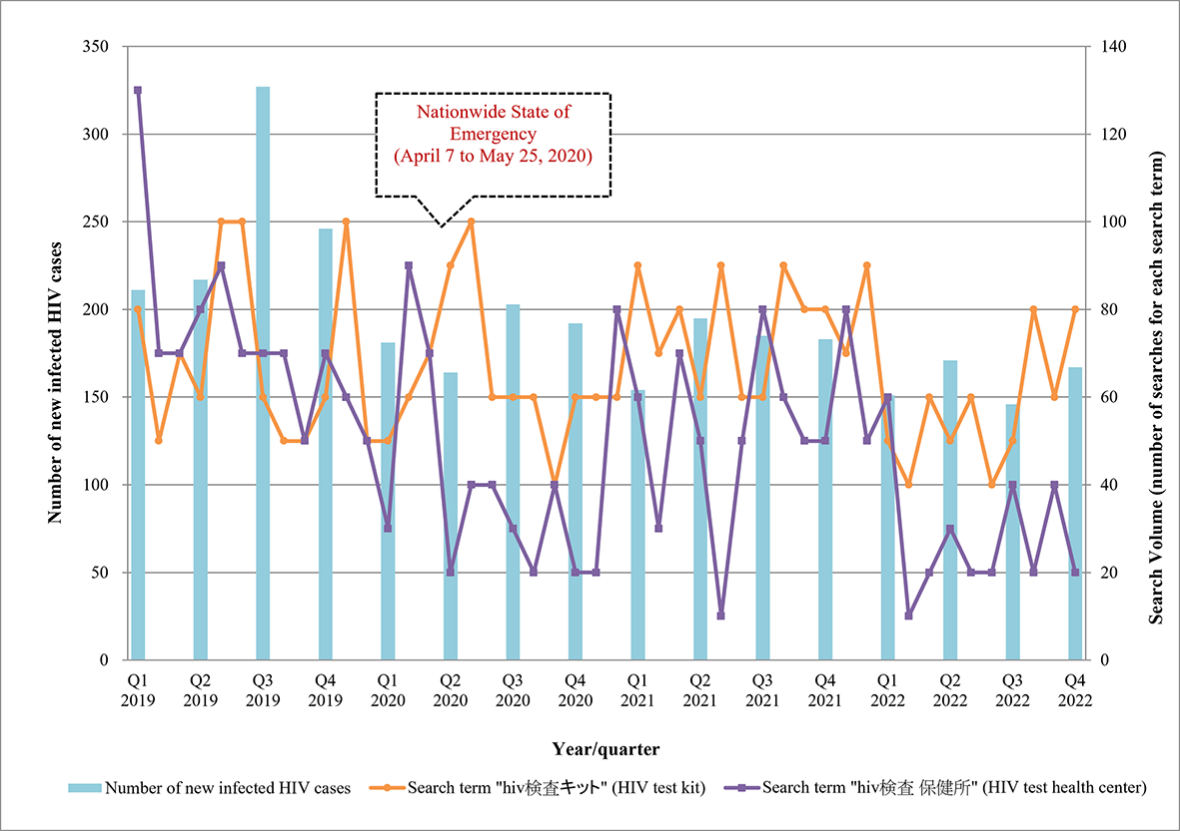
Monthly web-based search interest in HIV self-instructed and facility-based tests and quarterly number of new infected HIV cases from 2019 to 2022. The monthly search volumes of search terms ”HIV検査キット" (HIV test kit in Japanese, orange line) and "HIV検査 保健所” (HIV test health center in Japanese, purple line) from January 2019 to December 2022 according to the search engine provided by Yahoo! JAPAN are shown. The number of new HIV cases in the corresponding quarter, according to the AIDS Trend Committee Quarterly Report from the AIDS Prevention Foundation, API-Net AIDS Prevention Information Net, is shown with blue bars.

The quarterly number of newly infected HIV cases remained relatively stable since the declaration of a nationwide state of emergency concerning COVID-19, and the search interest remained high for self-testing relative to facility-based testing (Figure 3).

## Discussion

The analysis in our study illustrated that the web-based search interest in general HIV testing remained stable in Japan during the ongoing pandemic. Furthermore, compared to a decrease in HIV facility-based testing, the web-based interest in HIV self-testing has not changed in Japan. The findings suggest that a proportion of the population interested in getting an HIV test or obtaining relevant information may have a lower preference for HIV facility-based testing. This could imply that individuals might have opted for HIV self-testing during the COVID-19 pandemic.

A prolonged decreasing trend in the search volume of “HIV test health center” from late 2018 was observed in this study. This change in search volume was related to the real-world situation reported by recent publications that underscored the situation of facility-based HIV testing during the pandemic [7,8,23]. A trend analysis study exploring data from 2015 to the second quarter of 2020 (during the nationwide state of emergency in Japan) reported a significant decline in the number of HIV tests performed by public health centers in the second quarter of 2020 (n=9584 vs n=35,908 in Q2 2019) [7] and that this decrease coincided with the increase in the number of new HIV cases with an AIDS diagnosis in Japan [7]. Similar situations were reported in other countries, where an approximately 31% to 50% reduction in the number of HIV tests performed by public health centers was observed between 2019 and 2020 due to difficulty accessing testing facilities, shortage of medical staff for HIV-testing services, or closure of HIV-testing facilities [23,24].

Our search trend showed that the web-based search volume for HIV self-tests was relatively unaffected in Japan. Furthermore, we noted the trend for HIV-testing preference was more evident among male internet users. In Japan, men who have sex with men (MSM) constitute more than 70% of newly diagnosed people living with HIV [3,25]; therefore, the difference in search volume for different HIV-testing services most likely reflects the changing preferences among male internet users. This is consistent with the real-world situation reported by other countries. Investigators focusing on the high-risk population’s (MSM) HIV-testing behavior in China during the COVID-19 pandemic found the use of HIV self-testing increased compared to facility-based HIV testing, which decreased by more than 50% overall [26,27]. Earlier investigations in Sweden and Japan showed the interest and demand for HIV self-testing in high-risk populations such as MSM even before the COVID-19 pandemic [12,28]. A study in France also reported that the number of HIV self-testing kits sold in 2021 increased by 3% compared to 2020 [29]. Together, these publications further emphasized the preference for HIV self-tests for males in the ongoing COVID-19 pandemic and beyond.

In this study, interest in HIV self-testing has remained relatively high during the COVID-19 pandemic. Even though web-based search interest only indicates clinical information-seeking behavior, recent infodemiology studies, including that by Ornos et al [15], showed that search volume indices correlated positively with HIV prevalence and negatively with financial and health care service status. Another recent study in Japan by Ishimaru et al [30] also observed a positive correlation between internet search frequency for HIV/AIDS–related terms and the number of voluntary tests.

The change in search volume on HIV-testing preferences in Japan, which highlights the availability, accessibility, and regulatory approval status of HIV self-tests, may be imperative to reduce new HIV infection [7,8]. The pros and cons of alternative HIV-testing approaches such as the use of dried blood spot (DBS) test cards delivered by postal service have been investigated in Japan [8]. So far, DBS-based tests have not been approved as clinical samples for HIV testing in Japan; however, their use has steadily increased in the last two decades, most likely due to convenient and easy self-preparation of blood spots without the need to visit medical facilities [8]. Although the use of DBS is less sensitive than with a plasma sample, the feasibility and reliability of postal DBS have been preliminarily demonstrated in an outreach study in Japan [12]. Other HIV testing to be considered should include the introduction of HIV self-testing, DBS, or oral swabs as an alternative to plasma or serum specimens. However, since HIV self-testing is not yet approved by health authorities, individuals with positive test results may not have been referred to medical facilities for consultations after the test or received appropriate HIV care and treatment. This concern was illustrated in the study by Ejima et al [7], which reported that both the number of HIV tests and consultations performed by public health centers declined during the COVID-19 pandemic in Japan. We believe relevant supporting services, for instance, the provision of web-based counseling services and referral services after HIV self-testing, would be necessary to mitigate the potential concerns and negative impact of using HIV self-test kits without counseling and medical follow-up. Soon, the development of artificial intelligence chatbots may be used to provide real-time instruction and counseling for HIV self-testing users, which may offer potential solutions to this problem [31].

Several factors may limit the generalizability of the preliminary findings presented in this report. First, the data source was solely from Yahoo! JAPAN, and the internet searches conducted using other search engines were not included. Second, the data obtained from a web-based search engine may be subject to the nonrepresentative sampling or methodology bias inherent to the search platform. Third, there could be considerable differences in clinical information-seeking behavior between the internet user population and actual men and women who are affected by HIV; direct and causal relationships cannot be inferred from this study. Nonetheless, most previous studies using search engines have used Google Trends data, but in Japan, Yahoo! JAPAN is the most visited digital service in the country. In addition, sex and prefecture adjustment is possible with Yahoo! JAPAN data, making it appropriate to research topics likely affected by sex differences. Therefore, our preliminary infodemiology using the search engine provided by Yahoo! Japan Corporation is likely representative of the general trend in Japan.

Our infodemiology study indicated that there was a notable decrease in search volume for HIV facility-based testing during the COVID-19 pandemic. Further, a change in HIV-testing preference and interest in HIV self-testing was noted in Japan. To fully delineate and comprehend the changes in HIV-testing behavior, the situation should be continuously monitored and validated by clinical studies.

## Abbreviations

ART: antiretroviral therapy
DBS: dried blood spot
MSM: men who have sex with men
UNAIDS: Joint United Nations Programme on HIV and AIDS

## Data Availability

The data sets generated or analyzed during this study are not publicly available, complying with the Yahoo! Japan Corporation regulation, but they are available from the corresponding author upon reasonable request.
